# Combined association of clinical and lifestyle factors with non-restorative sleep: The Nagahama Study

**DOI:** 10.1371/journal.pone.0171849

**Published:** 2017-03-09

**Authors:** Takeshi Matsumoto, Yasuharu Tabara, Kimihiko Murase, Yoshimitsu Takahashi, Kazuya Setoh, Takahisa Kawaguchi, Shigeo Muro, Hiroshi Kadotani, Shinji Kosugi, Akihiro Sekine, Ryo Yamada, Takeo Nakayama, Michiaki Mishima, Fumihiko Matsuda, Kazuo Chin

**Affiliations:** 1 Department of Respiratory Medicine, Graduate School of Medicine, Kyoto University, Kyoto, Japan; 2 Center for Genomic Medicine, Graduate School of Medicine, Kyoto University, Kyoto, Japan; 3 Department of Health Informatics, School of Public Health, Kyoto University, Kyoto, Japan; 4 Department of Sleep and Behavioral Sciences, Shiga University of Medical Science, Shiga, Japan; 5 Department of Medical Ethics and Medical Genetics, School of Public Health, Kyoto University, Kyoto, Japan; 6 Center for Preventive Medical Science, Chiba University, Chiba, Japan; 7 Department of Respiratory Care and Sleep Control Medicine, Graduate School of Medicine, Kyoto University, Kyoto, Japan; Charité—Universitätsmedizin Berlin, GERMANY

## Abstract

**Background:**

Non-restorative sleep (NRS) was suggested to be associated with cardiovascular outcomes. However, causative factors for NRS have not been fully elucidated. This study aimed to clarify factors and their relationships with NRS to better understand the clinical and epidemiological implications of NRS and to develop a score that can objectively evaluate NRS status.

**Methods:**

Study subjects consisted of 9,788 community residents (age 53.6 ± 13.4 y). Subjective NRS as well as possible clinical and lifestyle factors for NRS were investigated by questionnaires. Other clinical parameters were obtained from personal records of information obtained at the baseline examination.

**Results:**

A total of 3,261 participants complained of NRS. Factors independently associated with subjective NRS were younger age (odds ratio = 1.43), use of a hypnotic drug (2.04), irregular sleep schedule (2.02), short sleep duration (<5 h, 11.7; 5–6 h, 4.81; 6–7 h, 2.40), frequent sleepiness (2.33), routine stress (4.63), no habitual exercise (1.61), nocturia symptoms (1.43), symptoms of gastroesophageal reflux disease (1.44), and depression (1.46) (all *P* <0.001). The NRS score comprised of these 10 factors was linearly associated with the frequency of subjective NRS (*P*_trend_ <0.001). Frequency of individuals with a high NRS score was greater in women (52.3%) than in men (42.1%, *P*<0.001), while no clear association was observed with common risk factors for cardiovascular diseases.

**Conclusions:**

NRS was a phenomenon representing various clinical and lifestyle features. Careful attention should be paid to individuals with a high NRS score who might be at risk for mental fatigue and have unfavorable lifestyle factors.

## Introduction

Both short and long sleepers have been reported to be at greater risk for mortality in various populations irrespective of cause of death [1, 2]. These associations might be independent of various risk factors that were potentially associated with poor outcomes, namely hypertension, type 2 diabetes, smoking, and lifestyle habits [3]. In addition to physically measured sleep duration, poor subjective sleep quality has also been suggested to be associated with systemic inflammation [4] and subclinical arterial diseases [5]. It was also reported that poor sleep quality might increase the risk of short sleep duration for the incidence of cardiovascular disease [6, 7].

Non-restorative sleep (NRS) is the subjective experience of unrefreshing sleep [8], and factors that have been strongly associated with NRS in association with insomnia were suggested to be short sleep duration, poor sleep quality, anxiety, and depressive symptoms [9]. Not only in patients with insomnia but also in the general population, a substantial number of individuals have experienced NRS, of which the frequency was estimated to be approximately 10% [10]. Given that NRS without insomnia was also significantly associated with poor sleep quality and psychological symptoms [10], NRS may represent potential risks for various clinical outcomes attributable to sleep problems even in the general population. However, factors involving NRS are largely unknown. In addition to findings on sleep parameters and psychological symptoms, a cross-sectional study in a Japanese general population [11], as well as our own study [12, 13], reported an involvement of unhealthy lifestyle factors and gastroesophageal reflux disease (GERD) symptoms in NRS.

Here, to clarify factors involved in NRS in a general population, as well as to develop a novel scoring system for evaluating the potential risk for NRS, we conducted a cross-sectional study by analyzing a dataset of the Nagahama Prospective Cohort for Comprehensive Human Bioscience (the Nagahama Study), which is a large-scale population-based cohort study in Japan. We also investigated a possible association of NRS with common clinical factors for cardiovascular diseases.

## Materials and methods

### Study participants

Study participants consisted of 9,788 middle-aged to elderly citizens who were participants in the Nagahama Study. The study cohort was recruited from 2008 to 2010 from the general population living in Nagahama City, a largely rural city of 125,000 inhabitants located in central Japan. Residents aged 30 to 74 years living independently in the community and with no physical impairment or dysfunction were recruited for the Nagahama cohort. The full dataset of the Nagahama cohort was used in this study.

All study procedures were approved by the ethics committee of Kyoto University Graduate School of Medicine and the Nagahama Municipal Review Board. Written informed consent was obtained from all participants.

### Subjective NRS

Subjective NRS was investigated by the following “yes-no” question: Do you get adequate rest during sleep? Individuals who answered “No” were considered to be experiencing NRS.

### Risk factors for NRS

The following factors potentially associated with NRS were evaluated by structured questionnaires: daily sleep, dietary and exercise habits, GERD symptoms, psychological factors (routine stress and depressive symptoms), and medication use.

We assessed sleep duration (On average, how many hours do you sleep per day?). We also assessed the regularity of the sleep schedule (Are your waking time and bed time regular?) and daytime sleepiness (How often do you feel sleepy?) as factors indicating daily sleep habits. Sleepiness was assessed by the following 3 responses: frequently, 3 or more days per week; rare, more than once a month; and never.

Unfavorable dietary habits were assessed by the following 4 questions with “yes-no” responses that were used in the public health checkup program in Japan: Do you skip breakfast 3 or more times per week?; Do you have dinner within 2 h before sleep 3 or more times per week?; Do you snack after dinner 3 or more times per week?; and Do you have a habit of eating rapidly? According to our previous reports [12, 13], any number of “Yes” answers were considered to indicate an unfavorable dietary habits score.

Exercise habit was investigated by a “yes-no” response to one question (Are you in the habit of exercising to sweat for over 30 minutes, 2 times weekly, for over a year?) according to the Japanese Exercise Guideline.

GERD symptoms were evaluated using the Frequency Scale for the Symptoms of GERD (FSSG), a well-validated and widely used questionnaire for the diagnosis of GERD [14]. Participants with an FSSG score of 8 or higher or those who were undergoing treatment of GERD were defined as having GERD.

Routine stress was investigated by a question (How often did you feel stress in the past year?) with 4 possible responses (frequently, sometimes, rarely, and never).

Depressive symptoms were assessed by the Mental Health Inventory [15]. Participants with a score ≤52 were considered as having depression. The cross-validity of this questionnaire in Japanese has been confirmed elsewhere [16].

Daily use of hypnotic or antipsychotic drugs including anti-anxiety and antidepressant drugs was determined by a questionnaire.

### Clinical parameters

Clinical parameters, including the plasma markers used in this study, were obtained from the personal records of participants who underwent baseline measurements for the Nagahama study.

### Statistical analysis

Values are expressed as mean ± standard deviation, median [interquartile range] or frequency. Group differences in continuous variables were assessed by an unpaired Student’s *t* test or Mann-Whitney U test, while those of categorical variables were assessed by a chi-squared test. Cochrane-Armitage test was used for a trend test. Factors independently associated with subjective NRS were assessed by a multivariate logistic regression analysis. Because short sleep duration is an established risk factor for NRS [9, 17], we performed a sensitivity analysis by excluding individuals who indicated a usual sleep duration shorter than 7h. To simplify the relationship between clinical and lifestyle factors and subjective NRS, the NRS score was calculated by summing the weighted number of independent risk factors with marked significance (*P* <0.01). The weighted score was determined based on the odds ratio in the regression model. A cut-off point, as well as its sensitivity and specificity, of the presently developed NRS score was obtained by a receiver operating characteristic (ROC) curves analysis. The data were also analyzed with the area under the curve. A two-tailed *P* value <0.05 was considered as statistically significant. All statistical analyses were performed using commercially available statistical software JMP 11.2.0 (SAS Institute Inc., Cary, NC, USA).

## Results

### Factors associated with subjective NRS

**Fig 1:**
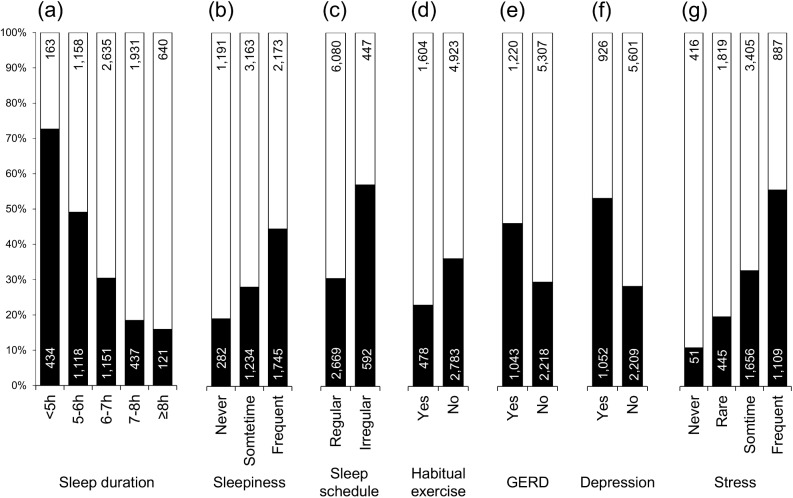
Frequency differences in subjective NRS. ■: non-restorative sleep (NRS); □: restorative sleep. (a) sleep duration, (b) sleepiness, (c) sleep schedule, (d) abitual exercise, (e) gastroesophageal reflux disease (GERD), (f) depression, (g) regular stress (all *P* <0.001). Number of individuals is shown within columns.

Clinical characteristics of study participants are summarized in Table 1. Factors markedly associated with subjective NRS are depicted in Fig 1. Frequency of NRS was significantly higher in individuals with short sleep duration, who experienced frequent sleepiness during daytime, or had an irregular sleep schedule. GERD symptoms and psychological factors were also associated with subjective NRS. In contrast, habitual exercise was identified as a protective factor for NRS. Other distinct characteristics of the NRS individuals were younger age, female sex, and frequent use of hypnotic and antipsychotic drugs (Table 2). Unfavorable dietary habits were also frequent in this group.

**Table 1:** Clinical characteristics of study participants.

			Total participants	Participants who slept ≥7h
N			(9,788)	(3,129)
Age (y)			53.6 ± 13.4	53.6 ± 14.5
Sex (male, %)			32.1	38.6
BMI (kg/m^2^)			22.3 ± 3.3	22.1 ± 3.3
Current smoker (%)			14.5	16.4
Frequent alcohol drinker (%)			22.7	26.6
Medication (%)	hypnotic drug		5.4	4.9
	antipsychotic drug		2.5	3.3
Sleep habit (%)	irregular sleep schedule		10.6	7.4
	sleep duration	<5h	6.1	-
		5-6h	23.2	-
		6-7h	38.7	-
		7-8h	24.2	75.7
		≥8h	7.8	24.3
	sleepiness	frequently	40.0	33.2
		rarely	44.9	47.3
		never	15.0	19.5
Stress (%)	frequently		20.4	16.7
	sometimes		51.7	50.4
	rarely		23.1	26.0
	never		4.8	6.9
No habitual exercise (%)			78.7	77.8
No. urinations during sleep time (%)*		0	44.3	37.9
		1	39.1	42.4
		≥2	16.6	19.7
Dietary habits (%)	skipping breakfast		9.2	9.0
	dinner within 2h before sleep		18.4	18.2
	snacking after dinner		20.9	16.5
	rapid eating		35.0	33.4
No. unfavorable dietary habits			0.8 ± 0.9	0.8 ± 0.9
GERD (%)			23.1	20.3
Depression (%)			20.2	16.3

Abbreviations: BMI, body mass index; GERD, gastroesophageal reflux disease.

Values are mean ± standard deviation or frequency. Participants who consumed alcohol 4 or more days per week were defined as frequent drinkers. Antipsychotic drugs included anti-anxiety drugs and antidepressant drugs.

* Data were available for 9,786 participants (participants who slept ≥7h, 3,128).

**Table 2:** Differences in clinical and lifestyle factors between those with RS and NRS.

		RS	NRS	*P*
N		(6,527)	(3,261)	
Age (y)		54.6 ± 13.5	51.5 ± 12.9	<0.001
Sex (male, %)		34.0	30.6	<0.001
BMI (kg/m^2^)		22.3 ± 3.2	22.3 ± 3.4	0.833
Current smoker (%)		13.9	15.6	0.021
Frequent alcohol drinker (%)		23.4	21.5	0.038
Medication (%)	hypnotic drug (%)	4.0	8.1	<0.001
	antipsychotic drug (%)	2.1	3.2	<0.001
No. urinations during sleep time (%)*				0.006
	0	43.4	46.2	
	1	40.2	36.9	
	≥2	16.4	16.8	
Dietary habits (%)	skipping breakfast	7.8	11.8	<0.001
	dinner within 2h before sleep	17.1	21.1	<0.001
	snacking after dinner	18.8	25.2	<0.001
	rapid eating	33.7	37.6	<0.001
No. unfavorable dietary habits		0.8 ± 0.9	1.0 ± 0.9	<0.001

Abbreviations: RS, restorative sleep; NRS, non-restorative sleep; BMI, body mass index.

Values are mean ± standard deviation or frequency.

* Data were available for 9,786 participants (RS, 6,526; NRS, 3,260).

To clarify factors independently associated with subjective NRS, multiple logistic regression analysis, including possible covariates as dependent variables, was performed (Table 3, Model 1). Age was included in the regression model as a dichotomic variable to obtain regression coefficient needed for the weighted score calculation with a cutoff of age 60 years [18]. However, even when age was included as a continuous variable, no substantial differences were observed in the results of the regression analysis (S1 Table). Results identified younger age, hypnotic drug use, poor sleep habits, sedentary lifestyle, nocturia symptoms, psychological symptoms, and GERD, but not unfavorable dietary habits, as independent major determinants of subjective NRS. Similar results were observed in the separate analysis of participants who slept ≥7h including 558 NRS individuals (Table 3, Model 2), whereas the positive associations of age, hypnotic drug use, and GERD became insignificant in this model. Unfavorable dietary habits were not identified as independent determinants even in the analysis that included individual factors in the regression model.

**Table 3:** Multivariate logistic regression analysis for subjective NRS.

		Model 1		Model 2	
		Total participants		Participants who slept ≥7h	
		OR (95% C.I.)	*P*	OR (95% C.I.)	*P*
Age (less than 60 y)		1.43 (1.27–1.61)	<0.001	1.21 (0.95–1.54)	0.116
Sex (men)		1.10 (0.94–1.26)	0.149	1.06 (0.82–1.37)	0.633
BMI (kg/m^2^)		0.99 (0.98–1.01)	0.236	0.98 (0.95–1.01)	0.173
Current smoking		1.08 (0.94–1.26)	0.275	1.16 (0.88–1.53)	0.296
Frequent alcohol drinker		1.01 (0.89–1.15)	0.871	0.90 (0.69–1.17)	0.430
Hypnotic drug		2.04 (1.65–2.52)	<0.001	1.50 (0.98–2.25)	0.059
Antipsychotic drug		0.88 (0.65–1.20)	0.416	1.10 (0.68–1.76)	0.684
Irregular sleep schedule		2.02 (1.74–2.35)	<0.001	2.02 (1.46–2.77)	<0.001
Sleep duration	<5h	11.7 (8.85–15.7)	<0.001		
	5-6h	4.81 (3.85–6.06)	<0.001		
	6-7h	2.40 (1.93–3.00)	<0.001		
	7-8h	1.28 (1.02–1.62)	0.035	1.31 (1.04–1.67)	0.022
	≥8h	Reference		Reference	-
Sleepiness	frequently	2.33 (1.99–2.74)	<0.001	2.42 (1.79–3.31)	<0.001
	sometimes	1.41 (1.20–1.66)	<0.001	1.33 (0.99–1.83)	0.063
	never	Reference		Reference	
Stress	frequently	4.63 (3.34–6.53)	<0.001	3.59 (2.07–6.55)	<0.001
	sometimes	2.57 (1.88–3.56)	<0.001	1.74 (1.05–3.07)	0.031
	rarely	1.57 (1.14–2.20)	0.005	1.11 (0.65–1.99)	0.723
	never	Reference		Reference	
No habitual exercise		1.61 (1.41–1.83)	<0.001	2.05 (1.54–2.77)	<0.001
No. urinations during sleep time	0	Reference		Reference	
	1	1.14 (1.02–1.27)	0.023	1.26 (1.00–1.59)	0.050
	2≤	1.43 (1.23–1.67)	<0.001	1.51 (1.12–2.04)	0.008
No. unfavorable dietary habits		1.03 (0.97–1.09)	0.331	1.13 (1.01–1.27)	0.038
GERD		1.44 (1.29–1.60)	<0.001	1.20 (0.95–1.50)	0.131
Depression		1.46 (1.29–1.65)	<0.001	1.56 (1.21–2.01)	<0.001

Abbreviations: NRS, non-restorative sleep; BMI, body mass index; GERD, gastroesophageal reflux disease; OR, odds ratio; C.I., confidence interval.

Participants whose data on urination were unavailable (Model 1, n = 2; Model 2, n = 1) were excluded from analysis.

### NRS score

**Fig 2:**
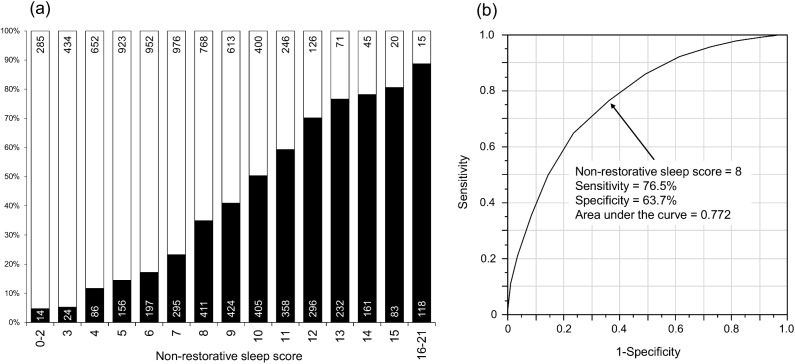
Frequency differences in subjective NRS according to the NRS score. ■: non-restorative sleep (NRS); □: restorative sleep. (a) frequency of NRS, (b) receiver operating characteristics curve for subjective NRS. Number of individuals is shown within columns.

For the components of the NRS score, 10 items were chosen by the result of the multivariate analysis (Table 3, Model 1 and Table 4). The weighted score was determined by assigning an appropriate point (nearest integral number) to each factor from the value of the regression coefficient in the logistic regression model [19] (S2 Table). For example, we took the value of a regression coefficient of 0.4 (corresponding to an odds ratio of 1.49) as 1 point, and the score for sleep duration <5h was taken as 6 points (2.4626 nearly equals 0.4*6). Frequency of subjective NRS was linearly and significantly (*P*_trend_ <0.001) associated with the NRS score (Fig 2A), and the ROC analysis indicated 8 points as the most sensitive and specific cut-off point for subjective NRS (Fig 2B).

**Table 4:** Weighted NRS score.

Variable	Category	Score
Age	≥60y/<60y	0/1
Hypnotic drug	no/yes	0/2
Irregular sleep schedule	no/yes	0/2
Sleep duration	≥7h/6-7h/5-6h/<5h	0/2/4/6
Sleepiness	never/sometimes/frequently	0/1/2
Habitual exercise	yes/no	0/1
Stress	never/rarely/sometimes/frequently	0/1/2/4
No. urinations during sleep time	0-1/≥2	0/1
GERD	no/yes	0/1
Depression	no/yes	0/1
Total		0–21

Abbreviations: NRS, non-restorative sleep; GERD, gastroesophageal reflux disease.

### NRS score and clinical parameters

**Fig 3:**
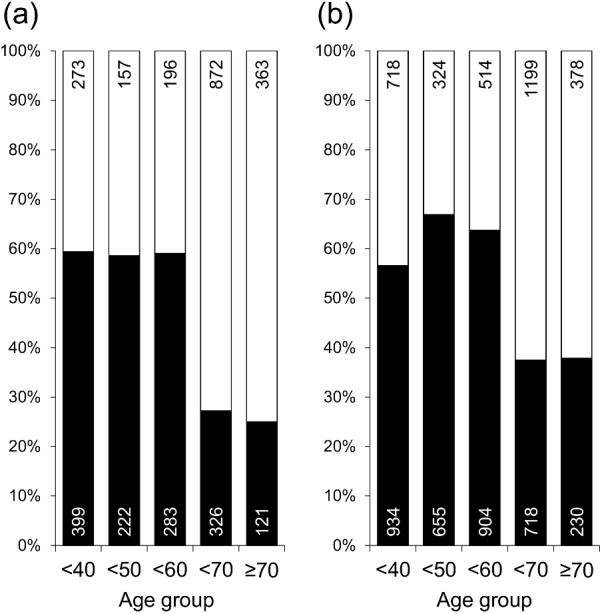
Frequency of participants with NRS score ≥8 points. Non-restorative sleep (NRS) score; ■: ≥8 points; □: <8 points. (a) men, (b) women. Number of individuals is shown within columns.

High NRS scores (≥8 points) were more frequent in women than in men (men, 42.1%; women, 52.3%; *P* <0.001) and in younger age groups than in older age groups (Fig 3). Table 5 summarizes clinical factors that differed significantly between subgroups divided according to NRS scores ≥8 points and <8 points. Although, in a crude analysis, significant group differences were observed in several clinical parameters, these associations became insignificant after adjustment for age and sex even in the sex-separated analysis.

**Table 5:** Differences in clinical parameters according to the NRS score.

	NRS score		*P*	
	≥8 points	<8 points	Crude	Adjusted
N	(4,792)	(4,994)		
SBP (mmHg)	121.1 ± 17.0	125.6 ± 18.2	<0.001	0.073
DBP (mmHg)	75.1 ± 11.3	76.7 ± 11.3	<0.001	0.750
Glucose (mg/dl)	89.5 ± 17.1	91.3 ± 15.2	<0.001	0.864
HbA1c (%)	5.44 ± 0.55	5.49 ± 0.54	<0.001	0.062
LDL cholesterol (mg/dl)	121.8 ± 31.6	123.9 ± 30.6	0.001	0.268
HDL cholesterol (mg/dl)	66.1 ± 17.2	64.4 ± 16.8	<0.001	0.469
CRP (ng/dl)	272 [124–596]	305 [144–669]	<0.001	0.589

Abbreviations: NRS, non-restorative sleep; SBP, systolic blood pressure; DBP, diastolic BP; HbA1c, hemoglobin A1c; LDL, low-density lipoprotein; HDL, high-density lipoprotein; CRP, high-sensitive C-reactive protein.

Values are mean ± standard deviation or median [interquartile range]. Adjusted factors were age and sex.

## Discussion

We presently clarified factors that were significantly associated with subjective NRS. Not only sleep parameters but also clinical and psychological factors, namely GERD symptoms, depressive mood, routine stress, nocturia symptoms, and hypnotic drug use, as well as lifestyle factors such as lack of an exercise habit, were significantly associated with subjective NRS. The NRS score calculated as sum of these 10 items was significantly higher in women and in younger age group, whereas no clear association was observed with other clinical factors.

Frequency of subjective NRS was 33.3% in our study population, while it was reported to vary according to the study population and the methods used for the NRS assessment [9–11, 20–22]. Another general-population based study in Japan [11] in which the mean age was slightly older than in our study population reported that the frequency of NRS was 19.2% in men and 26.3% in women using the same single “yes-no” question that we used. Other studies using different methods in the assessment of NRS reported frequencies of NRS among community residents of 25.2% to 43.0% in United States [9, 22], 2.4 to 16.1% in several European cohorts [10], 7.9% in Finland [21], and 4.7% in South Korea [20]. Thus, the frequency of NRS might be different among populations, and Asians might be at risk for NRS due to a tendency to have risk factors for NRS, namely short sleep duration [23, 24] and depressive symptoms [25, 26]. However, as no clear regional specificity was observed in the results of previous studies, these issues deserve further investigation using the same assessment tool for NRS. The NRS score developed in this study might be a good objective method to assess population differences in the frequency of NRS.

Factors previously identified as risk factors for subjective NRS ranged over a wide variety of areas, e.g., younger age [10, 11, 22, 27], women [22, 27], short sleep duration [9, 17], physical functional decline [9], routine stress [10], and depressive symptoms [9, 10]. A strength of this study is therefore the comprehensive analysis of these previously noted risk factors in a single population and clarifying independent associations of these risk factors with subjective NRS. Among the risk factors, short sleep duration had the highest odds ratio for NRS. Results of a longitudinal study that reported a higher incidence of NRS in short sleepers [17] further support an adverse impact of short sleep duration. Although one previous study [10] reported an opposite association, namely a lower NRS risk in short sleepers, that result might have been due to including extra weekend sleep time in the multivariate analysis; i.e., short sleepers are likely to get extra sleep on their days off and that extra sleep time could be positively associated with a higher frequency of NRS. Routine stress was a second independent factor for NRS. It is well known that perceived stress is one risk factor for poor sleep quality [28], and the association between routine stress and NRS might partially develop via poor sleep quality.

In a sub-analysis excluding subjects with sleep duration <7h, sleep parameters and psychological factors remained significant determinants for NRS while the association with age became insignificant. Since mean age, as well as the odds ratio of age as a continuous variable for NRS, was not substantially different between the analysis using the total population and that using participants who slept ≥7h, the loss of significance might be due to the decreased number of study participants and consequently low statistical power. The positive association of hypnotic drug use and GERD symptoms with NRS also became insignificant, possibly due to the higher frequency of these factors in short sleepers [12].

No marked association was observed between the NRS score and major clinical factors related to common diseases even in the sex-separated analysis whereas NRS has been suggested to be associated with the incidence of heart failure [29] and fatal injuries [30] in general populations, poor prognosis in patients with coronary disease [31], and all-cause mortality in a male general population [32]. The adverse effect of NRS might therefore be mediated via other pathophysiological pathways independent of common diseases. Results of a previous longitudinal study [6] that reported a higher incidence of cardiovascular outcomes in short sleepers or that common risk factors were not different between middle-to-long sleepers and short sleepers support our considerations.

We developed an objective score for NRS with the preferable cut-off point of 8. Given that this score consists of all factors that were identified as independent determinants for NRS in this comprehensive analysis, as well as the linear correlation of the NRS score with the frequency of subjective NRS, the newly developed NRS score may be reliable to identify individuals with potential NRS. Although further studies are required to clarify the clinical and epidemiological significance of NRS, this score may be useful in a cohort study or an interventional study to objectively evaluate intra-individual differences in NRS status.

We recognize several limitations in this study. First, as this study was cross-sectional, a causal relationship between subjective NRS and the related factors remains to be elucidated. Second, we did not evaluate sleep apnea syndrome because it is difficult to objectively assess the disease conditions without using polysomnography or the oxygen desaturation index. Given that sleep apnea is a common phenomenon in general populations [33, 34] and has been suggested to be a cause of NRS [35], a possible involvement of sleep apnea in NRS deserves further investigation. However, as the NRS score includes daytime sleepiness as a factor, this score may indirectly evaluate the potential effect of sleep apnea on NRS. Third, we could not perform evaluations using a more specific questionnaire such as the Hospital Anxiety and Depression Scale, Epworth Sleepiness Scale, or the Restless Legs Severity Scale.

In conclusion, although there were several limitations in this study, we clarified factors for subjective NRS in a large-scale general population and, based on those findings, developed a score that can objectively evaluate NRS. Results of the present study may help to further understand the clinical and epidemiological importance of NRS. The NRS score can be utilized in public health monitoring of NRS that may be accompanied by mental fatigues and unfavorable lifestyle factors.

## Supporting information

S1 Table Multivariate logistic regression analysis for subjective NRS. Abbreviations: NRS, non-restorative sleep; BMI, body mass index; GERD, gastroesophageal reflux disease; OR, odds ratio; C.I., confidence interval. Participants whose data on urination were unavailable (Model 1, n = 2; Model 2, n = 1) were excluded from analysis.
(DOCX)

S2 Table Regression coefficient from the multivariate logistic regression model. Abbreviations: GERD, gastroesophageal reflux disease.
(DOCX)
