# Supplementary material for: Combined association of clinical and lifestyle factors with non-restorative sleep: The Nagahama Study
Source: PLoS One. 2017 Mar 9;12(3):e0171849. doi: 10.1371/journal.pone.0171849 (PMC5344328; doi:10.1371/journal.pone.0171849)
Supplement: S1 Table — Abbreviations: NRS, non-restorative sleep; BMI, body mass index; GERD, gastroesophageal reflux disease; OR, odds ratio; C.I., confidence interval. Participants whose data on urination were unavailable (Model 1, n = 2; Model 2, n = 1) were excluded from analysis. (DOCX) [file pone.0171849.s001.docx]

**S1 Table. Multivariate logistic regression analysis for subjective NRS**

|  | | Model 1 | | Model 2 | |
| --- | --- | --- | --- | --- | --- |
|  | | Total participants | | Participants who slept ≥7h | |
|  | | OR (95% C.I.) | *P* | OR (95% C.I.) | *P* |
| Age (1 y) | | 0.99 (0.99-1.00) | <0.001 | 0.99 (0.99-1.00) | 0.169 |
| Sex (men) | | 1.08 (0.95-1.22) | 0.226 | 1.06 (0.82-1.37) | 0.645 |
| BMI (1 kg/m^2^) | | 0.99 (0.98-1.01) | 0.248 | 0.98 (0.95-1.01) | 0.195 |
| Current smoking | | 1.09 (0.94-1.26) | 0.257 | 1.16 (0.88-1.53) | 0.301 |
| Frequent alcohol drinker | | 1.02 (0.89-1.16) | 0.791 | 0.90 (0.70-1.17) | 0.442 |
| Hypnotic drug | | 1.97 (1.60-2.43) | <0.001 | 1.51 (0.99-2.26) | 0.057 |
| Antipsychotic drug | | 0.90 (0.66-1.22) | 0.486 | 1.12 (0.68-1.78) | 0.656 |
| Irregular sleep schedule | | 2.00 (1.72-2.33) | <0.001 | 2.00 (1.45-2.75) | <0.001 |
| Sleep duration | <5h | 12.1 (9.14-16.2) | <0.001 |  |  |
|  | 5-6h | 4.96 (3.96-6.24) | <0.001 |  |  |
|  | 6-7h | 2.45 (1.97-3.07) | <0.001 |  |  |
|  | 7-8h | 1.30 (1.03-1.65) | 0.026 | 1.33 (1.05-1.69) | 0.018 |
|  | ≥8h | Reference |  | Reference | - |
| Sleepiness | frequently | 2.35 (2.01-2.77) | <0.001 | 2.41 (1.78-3.30) | <0.001 |
|  | sometimes | 1.42 (1.21-1.67) | <0.001 | 1.33 (0.98-1.82) | 0.067 |
|  | never | Reference |  | Reference |  |
| Stress | frequently | 4.86 (3.51-6.85) | <0.001 | 3.62 (2.09-6.60) | <0.001 |
|  | sometimes | 2.67 (1.96-3.70) | <0.001 | 1.75 (1.05-3.09) | 0.030 |
|  | rarely | 1.61 (1.17-2.25) | 0.003 | 1.11 (0.65-2.00) | 0.704 |
|  | never | Reference |  | Reference |  |
| Habitual exercise | | 0.61 (0.54-0.70) | <0.001 | 0.49 (0.36-0.65) | <0.001 |
| No. urinations during sleep time | 0 | Reference |  | Reference |  |
|  | 1 | 1.11 (0.99-1.24) | 0.064 | 1.26 (1.00-1.59) | 0.053 |
|  | 2≤ | 1.35 (1.16-1.57) | <0.001 | 1.50 (1.11-2.04) | 0.009 |
| No. unfavorable dietary habits | | 1.04 (0.98-1.10) | 0.225 | 1.13 (1.01-1.27) | 0.039 |
| GERD | | 1.45 (1.29-1.61) | <0.001 | 1.20 (0.95-1.51) | 0.122 |
| Depression | | 1.46 (1.29-1.66) | <0.001 | 1.55 (1.20-2.00) | <0.001 |

Abbreviations: NRS, non-restorative sleep; BMI, body mass index; GERD, gastroesophageal reflux disease; OR, odds ratio; C.I., confidence interval.

Participants whose data on urination were unavailable (Model 1, n = 2; Model 2, n = 1) were excluded from analysis.
