# Supplementary material for: Combined association of clinical and lifestyle factors with non-restorative sleep: The Nagahama Study
Source: PLoS One. 2017 Mar 9;12(3):e0171849. doi: 10.1371/journal.pone.0171849 (PMC5344328; doi:10.1371/journal.pone.0171849)
Supplement: S2 Table — Abbreviations: GERD, gastroesophageal reflux disease. (DOCX) [file pone.0171849.s002.docx]

**S2 Table. Regression coefficient from the multivariate logistic regression model.**

| Variable | | Regression coefficient |
| --- | --- | --- |
| Age (less than 60 y) | | 0.3592 |
| Hypnotic drug | | 0.7133 |
| Irregular sleep schedule | | 0.7047 |
| Sleep duration | ≥8h | Reference |
|  | 6-7h | 0.8743 |
|  | 5-6h | 1.5709 |
|  | <5h | 2.4626 |
| Sleepiness | never | Reference |
|  | sometimes | 0.3458 |
|  | frequently | 0.8454 |
| No habitual exercise | | 0.4736 |
| Stress | never | Reference |
|  | rarely | 0.4528 |
|  | sometimes | 0.9423 |
|  | frequently | 1.5325 |
| No. urinations during sleep time | 0 | Reference |
|  | ≥2 | 0.3576 |
| GERD | | 0.3614 |
| Depression | | 0.3773 |

Abbreviations: GERD, gastroesophageal reflux disease.
